# Dense encoding of natural odorants by ensembles of sparsely activated neurons in the olfactory bulb

**DOI:** 10.1038/srep36514

**Published:** 2016-11-08

**Authors:** Olivier Gschwend, Jonathan Beroud, Roberto Vincis, Ivan Rodriguez, Alan Carleton

**Affiliations:** 1 Department of Basic Neurosciences, Faculty of Medicine, University of Geneva, 1 rue Michel-Servet, 1211 Genève 4, Switzerland; 2 Geneva Neuroscience Center, University of Geneva, Switzerland; 3 Department of Genetics and Evolution, University of Geneva, 1211 Geneva, Switzerland

## Abstract

Sensory information undergoes substantial transformation along sensory pathways, usually encompassing sparsening of activity. In the olfactory bulb, though natural odorants evoke dense glomerular input maps, mitral and tufted (M/T) cells tuning is considered to be sparse because of highly odor-specific firing rate change. However, experiments used to draw this conclusion were either based on recordings performed in anesthetized preparations or used monomolecular odorants presented at arbitrary concentrations. In this study, we evaluated the lifetime and population sparseness evoked by natural odorants by capturing spike temporal patterning of neuronal assemblies instead of individual M/T tonic activity. Using functional imaging and tetrode recordings in awake mice, we show that natural odorants at their native concentrations are encoded by broad assemblies of M/T cells. While reducing odorant concentrations, we observed a reduced number of activated glomeruli representations and consequently a narrowing of M/T tuning curves. We conclude that natural odorants at their native concentrations recruit M/T cells with phasic rather than tonic activity. When encoding odorants in assemblies, M/T cells carry information about a vast number of odorants (lifetime sparseness). In addition, each natural odorant activates a broad M/T cell assembly (population sparseness).

Odor representations undergo substantial changes across the consecutive layers of the olfactory network. For instance, evoked inputs elicited in olfactory sensory neurons (OSNs) undergo significant reshaping by the recurrent and lateral inhibition of interneurons in the olfactory bulb (OB)^1^,^2^,^3^. This inhibition was initially proposed to tune M/T cell activity with a dense center-surround inhibition regime^4^,^5^. But more recent work revised this idea and suggested that M/T cells actually receive inputs from sparsely distributed glomeruli^6^, consequently resulting in sparse and narrowly tuned M/T output curves^7^. Based on these results, it was concluded that M/T cells display both a large population sparseness (i.e. fraction of neuron responding to a particular stimulus) and a large lifetime sparseness (i.e response selectivity of a neuron to different stimuli)^8^. However, since these studies were conducted in anesthetized mice and since it is now well admitted that activity in an awake state strongly differs from the one observed during anesthesia^9^,^10^, definitive conclusions about M/T sparseness must only be made after performing experiments in awake mice.

Recent works proposed that sparsening of M/T cell firing drives GABAergic-dependent pattern separation of odorant representations^10^,^11^,^12^,^13^ and might thereof be dependent on weak though highly informative temporal changes of spiking in M/T cell ensembles rather than on single neuron tonic changes. In regards to those results and considering that natural odorants activate dense glomerular patterns at their intrinsic concentrations^14^, we hypothesized that M/T cells may be more broadly tuned in awake animals than previously shown in anesthetized animals^6^,^7^.

Using a combination of optical imaging and tetrode recordings in awake head-restrained mice, we monitored the glomerular and M/T cells responses to a large set of natural odorants. As M/T cells encode odorant information with subtle phasic temporal changes of spiking^9^,^15^,^16^, we developed an analysis based on temporal patterning of population activity. Natural odorants at their native concentrations recruited a large fraction of M/T cells that likely reflect the dense glomerular maps evoked by these odorants^14^. Decreasing odorant concentration reduced the number of activated glomeruli and increased the selectivity of M/T cells. We conclude that M/T ensembles process natural odorants with a denser representation than previously observed with monomolecular odorant, the density of the code being adapted to the density of the incoming input patterns.

## Results

### M/T cell responses to native concentrations of natural odorants

**Figure 1:**
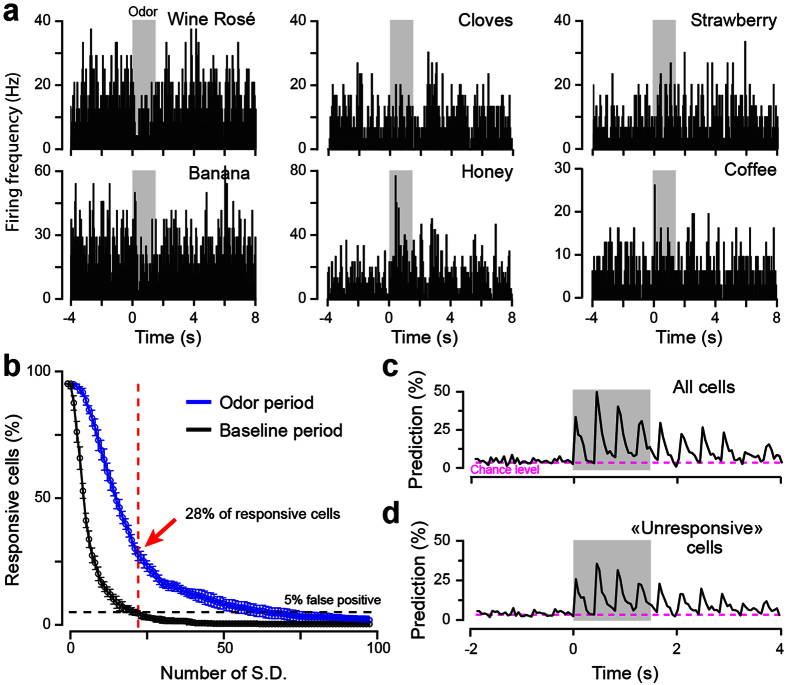
M/T cell spiking responses to natural odorants. (**a**) Representative examples of peristimulus time histograms (PSTH, 50 ms bins) evoked by six different natural odorants at their natural concentration in different M/T cells (all different). (**b**) Percentage of cells responding to at least one odorant. A cell is considered as responsive when a significant difference between baseline and odor cycles firing is observed, taking into account the baseline variance (i.e. standard deviation, S.D.; see Methods). We set the number of S.D. in order to have no more than 5% false responding neurons in the baseline. (**c,d**) Prediction performances, calculated based on population activity (every 50 ms), for a set of 29 natural odorants using the complete cell population (81 cells, **c**) or using only the “unresponsive” cells as defined in (**b**) (58 cells, **d**). Grey boxes indicate odor application. Dashed lines indicate the prediction chance level (i.e. 3.45%, 1/29 odorants). Oscillations represent changes in classifier performances following sniff-driven M/T cells activity.

We selected a large set of natural odorants (*n* = 29) at concentrations encountered when an animal is close to the odor source^14^ (referred as high concentrations hereafter). These concentrations have previously been shown to activate dense glomeruli patterns (see for quantifications^14^). We performed tetrode recordings in the olfactory bulb of awake head-restrained mice^9^,^11^,^17^,^18^ exposed to natural odorants, and isolated individual M/T cells (*n* = 81 M/T cells from 4 mice). As previously described^9^, M/T cells responded to the stimuli with weak and phasic firing rate changes, as observed on the peristimulus time histograms (50 ms binned PSTH; Fig. 1a). To estimate the selectivity of response of single M/T cells, we performed a receiver operating characteristic (ROC) analysis^15^ by comparing the spiking activity over the first respiratory cycle following odor application to the baseline firing and extracted the number of responding cells by recurrently increasing the area under the ROC curve (auROC). For an auROC of 0.75 and 0.9 (i.e. 25% and 10% of false positive, respectively), the percentage of responding odor-cell pairs was 45.3% and 16.9% respectively (Fig. S1).

As most M/T cells did not exhibit tonic changes^9^, we also identified responding neurons by analyzing the phasic activity across each sniff 6,9,15. We therefore used a method that takes into account the redistribution of spike timing over respiratory cycles by comparing baseline and odor epochs (see for details^9^). To estimate the number of false positives detected by our method, we also performed a control analysis by similarly comparing two baseline epochs (see methods). We next set a cutoff threshold for which we detected no more than 5% false positive responses in the baseline period. By comparing the baseline and the odor-evoked epochs, we found that ~28% of the cells changed their firing properties for at least one odor (Fig. 1b), a percentage that is higher than the number of responding cells determined by the auROC analysis. Yet, previous work from our laboratory showed that M/T cells that are considered as unresponsive are still informative when using a simple population-based linear classifier^9^. Confirming this previous observation, we found that ensemble activity (binned with 8 bins of 50 ms per breath) predicts natural odor identity, reaching a maximum of 49% accurate prediction (chance level value: ~3%, Fig. 1c). However, when the responsive neurons (identified using the analysis in Fig. 1b) were removed from the complete sampled population (i.e. leaving only supposedly “unresponsive” cells), the maximum classification performance still remained above chance level while decreasing to 36% (Fig. 1d). These results indicate that our method based on single cell activity is still insufficient to accurately separate informative from non-informative cells. In light of these results, we developed an alternative method better suited to identify informative M/T neurons.

### M/T cells are broadly tuned to natural odorants

**Figure 2:**
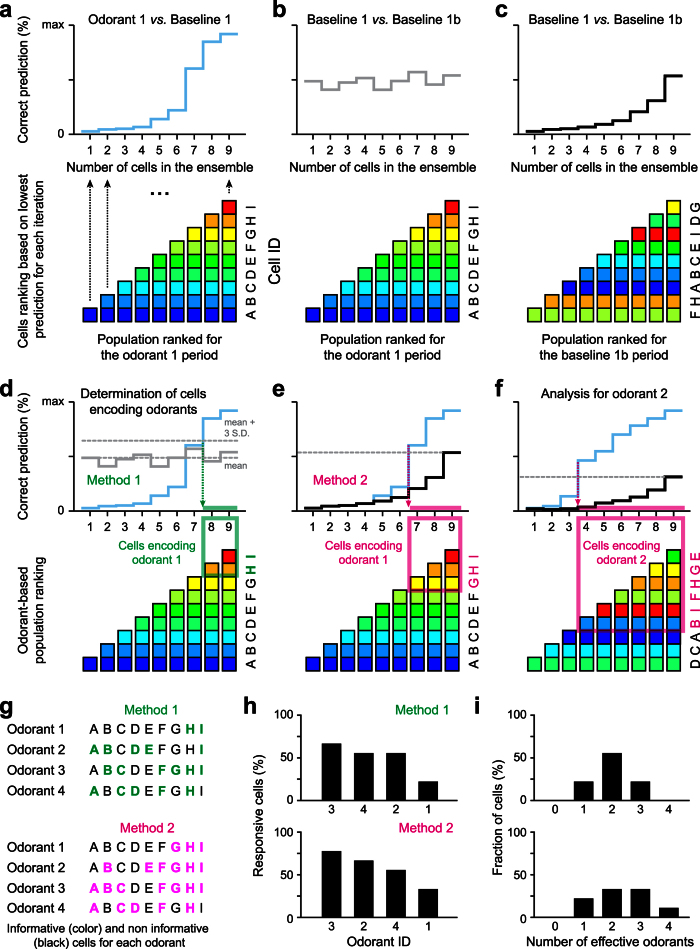
Method used to identify odor-responsive M/T cells based on their contribution to population coding. (**a**) For each odorant, we used a population prediction algorithm to compare activity between baseline and odor epochs as a function of the number of cells in the assembly (*blue curve*). We started with the cell displaying the smallest prediction (cell A in the example) and then progressively added the next cells which least improved the prediction performance (cell B in the example) until all cells are ranked in the ensemble according to how strongly they contribute to the discrimination between odor and baseline epochs (in the example, ranking is ABCDEFGHI for odorant 1). (**b**) As a control, we ran the classification algorithm on two different parts of the baseline and used the same ranking sequence as in (**a**). (**c**) As an alternative control, we compared again two different parts of the baseline but repeated the cell ranking procedure discussed in **a** on the baselines (in the example, the ranking order on the baseline is FHABCEIDG). (**d,e**) To identify which neurons contribute to the population code and therefore encode odorants, we used the odor prediction curve and defined a cut-off threshold above which neurons are defined to carry odorant information (green and magenta boxes). We used two methods that differed in conservatism. (**d**) For the first method (most conservative), the cutoff was the mean +3 S.D. of the baseline prediction curve in (**b**). In the example, cells responding to odorant 1 are neurons H and I. (**e**) For the second method, the cutoff was the maximum of baseline prediction curve in (**c**). (e.g. cells responding to odorant 1 are G, H and I). (**f**) The entire procedure was repeated for each odorant independently. In the example, for odorant 2, the ranking is DCABIFHGE and the responsive neurons are B, I, F, H, G, and E. (**g**) For each odorant, the responding neurons were identified and color coded. (**h**) Fraction of responding cells plotted for each odorant and for the two methods. (**i**) Fraction of the cell population responding to odorants for both methods.

**Figure 3:**
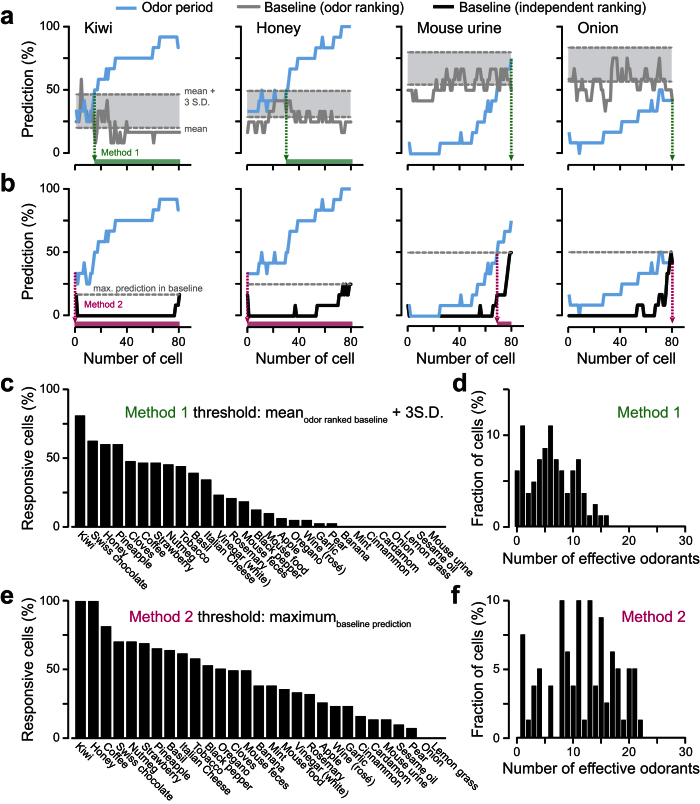
M/T cells are broadly tuned at high concentrations of natural odorants. **(a,b)** Examples of classification performances for 4 different natural odorants computed by comparing the population activity between baseline and odor epochs as a function of the number of cells in the assembly (*blue curves*). (**a**) The grey curves represent the classification performance computed on another part of the baseline (internal control) using the same cell order. To identify which neurons are responding to odorants, we used the mean plus three standard deviations as the cut-off threshold (*gray dotted line*; method 1). The number of responding cells for each odorant is pointed by the green arrows and boxes. (**b**) In a second method, we ranked the cells in the baseline independently and defined the maximum of the curve as the cut-off threshold. The number of responding cells is indicated by the magenta arrows and boxes. (**c,e**) Fraction of responding cells plotted for each odorant and computed with methods 1 (**c**) and 2 (**e**). (**d,f**) Fraction of population plotted as a function of the number of effective odorants computed with methods 1 (**d**) and 2 (**f**).

To identify which neuron was informative about a particular stimulus, we developed an algorithm (based on a simple classifier) that tested whether specific neuronal ensembles could classify odorant identity. For each stimulus, we used our algorithm to distinguish the activity of the baseline from the odor epochs (Figs 2 and 3). We started by computing the classifier with the smallest ensemble (i.e. a single neuron) displaying the lowest predictive performance. We then added to the ensemble the activity of the neuron which least improved the performance of the classifier. By increasing the number of neurons using the same recurrence, we ranked all cells in the ensemble according to their contribution in predicting each odor epoch from their respective baseline epochs (*blue curves* in Figs 2a and 3a,b). In order to avoid potential wrong prediction caused by noise, we defined a cutoff threshold below which cells were considered as false positives. To set this threshold, we used two different methods (Figs 2 and 3). First, we considered the sequence of cells obtained by the recurrent analysis to classify different parts of the baseline. We further defined the mean of the performance curve plus two (Fig. S2) or three (Fig. 3a) standard deviations as a cutoff threshold (*grey curves* in Figs 2b and 3a). For the second method, we performed the same recurrent cell ranking procedure by comparing and predicting two baseline epochs (*black curve*s in Figs 2c and 3b). In this case, the maximum of the baseline prediction curve was defined as a cutoff. For both methods, the part of the odorant-specfic ranking curve above the cutoff represented the cell ensemble carrying information about the considered odorant (Figs 2a–d and 3a,b). In order to compute a tuning profile, the entire procedure was repeated independently for the 29 natural odorants (Figs 2d–f and 3a,b).

For some odorants, both methods identified responding neurons. For example, most neurons (First method: 66 and 55 cells for kiwi and honey respectively; Second method: 81 cells for both odorants) carried information about honey and kiwi, while onion and mouse urine were weakly or not encoded by the set of recorded cells (Fig. 3a,b). We then computed a tuning curve showing the fraction of cells that carried information to each of the 29 natural odorants (Fig. 3c,e). Most odorants triggered informative activity in the recorded population (72 and 93% responding odorants using Methods 1 and 2, respectively). We further plotted the fraction of responding cells as a function of the number of effective odorants and we observed that individual M/T cells were encoding ~23–43% of the tested odorants (12.5 ± 5.8 odorants and 6.8 ± 2.9 for methods 1 and 2, respectively; Fig. 3d,f).

Taken together, these results suggest that the spike temporal envelope of M/T ensembles evoked by odors is sufficient to carry information about a large fraction of odorants. Hence, the dense representation of natural odorants previously observed at the level of the glomerular layer^14^ is maintained within the M/T cell population.

### Sparsening of M/T cells tuning at low concentrations of natural odorants

**Figure 4:**
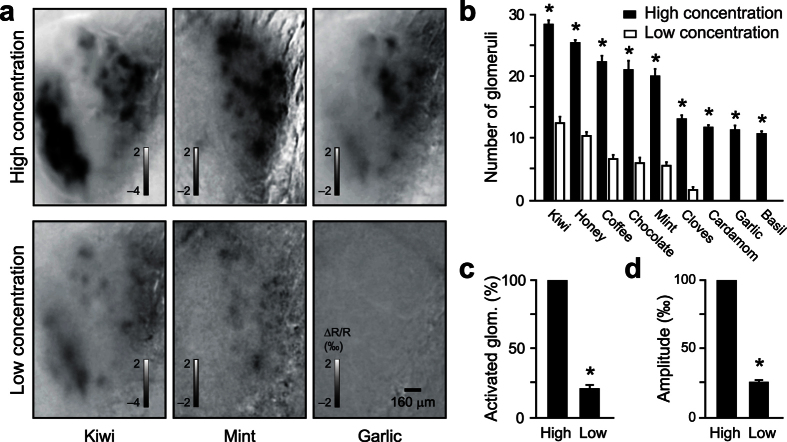
Patterns of activated glomeruli vary in density with natural odorant concentrations. (**a**) Glomerular activity patterns evoked by three natural odorants at high and low concentrations in the same mouse. The images represent the average of all frames during odor application. (**b**) Number of glomeruli activated by each natural odorant tested at high and low concentrations (*n* = 3 mice). High concentrations systematically activate more glomeruli (repeated measure ANOVA, *P* = 2.10^−4^, *post-hoc Newman-Keuls test at least *P* < 5.10^−4^). (**c**) Number of glomeruli activated by all odorants normalized to the number of glomeruli activated at high concentrations for each odorant (Low: 20.7 ± 3.4%, number of activated glomeruli: High = 19 ± 1 and Low = 5 ± 1, *n* = 24 mouse/odor pairs, *Wilcoxon test *P* < 10^−5^). (**d**) Amplitude of the activated glomerular response normalized to the amplitude calculated for high concentration (25.9 ± 2.4%, *n* = 24 mouse/odor pairs, *Wilcoxon test *P* < 10^−4^). Data are presented as mean ± s.e.m.

M/T cell responses have been previously reported to be highly selective (i.e. low lifetime sparseness)^6^,^7^. If we exclude the effect of anesthetics on coding properties^9^,^14^, one possible additional explanation for this discrepancy with our results may be due to the odorant concentrations used. We hence evaluated whether a sparser odor map induced at lower concentration would result in highly selective M/T cell tuning. To test this possibility, we first studied how stimulus strength would impact the representation of natural odorants in the mouse olfactory bulb (OB). Using intrinsic optical signal (IOS) imaging^14^,^19^,^20^,^21^,^22^, we imaged the patterns of activated glomeruli evoked by different natural odorants in awake and head-restrained mice (Fig. 4a). Odorants at high concentration (*n* = 8) activated a large number of glomeruli in all animals tested (*n* = 3 mice, Fig. 4a,b), consistent with our previous findings^14^. At concentrations corresponding to a 1:50 dilution (referred as low concentration hereafter), we observed a global and significant sparsening of activated glomeruli patterns (Fig. 4a,b). On average, the number of activated glomeruli was reduced by fivefold (Fig. 4c) and the amplitude of glomerular responses was reduced by fourfold (Fig. 4d). In summary, varying odorant concentrations evoked glomeruli patterns that varied in density and strength.

**Figure 5:**
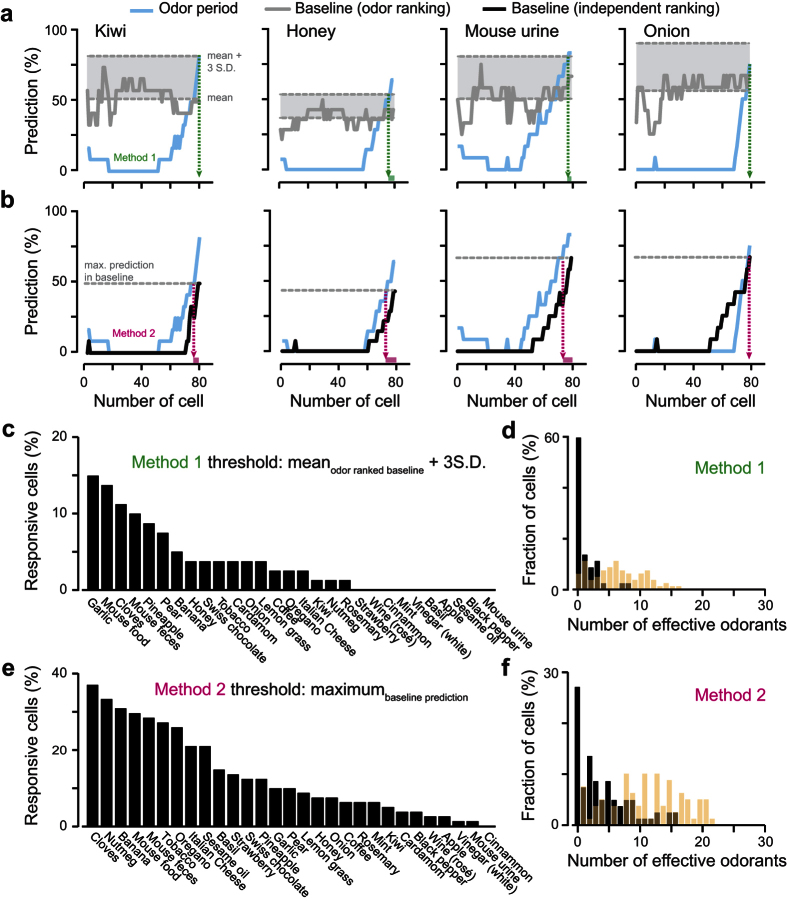
Sparsening of M/T cells tuning at low concentration of natural odorants. (**a,b**) Examples of classification performances for four natural odorants (same as in Fig. 3) presented at low concentrations. Same procedure and code as described for Fig. 3. (c,e) Fraction of responding cells plotted for each odorant and computed with methods 1 (**c**) and 2 (**e**). (**d,f**) Fraction of the cell population plotted as a function of the number of effective odorants computed with methods 1 (**d**) and 2 (**f**). Colored histogram corresponding to the tuning profile found at higher concentrations (same as Fig. 2) are plotted for comparison.

Since a 50-fold dilution induced a robust sparsening of glomerular inputs, we aimed at recording another set of M/T cells in awake head-restrained mice (*n* = 79 M/T cells from 4 mice) using the same 50-fold diluted natural odorants. Though M/T cell population activity still predicts natural odor identity at this concentration, the maximum performance was much lower than the one observed at high concentrations (Fig. S3). In addition, the number of informative M/T cells was reduced when lowering odorant concentrations (compare Figs 3a–d and 5a–d), suggesting a sparsening of odorant representations. In this case, M/T cells responded to 4–5 times less odorants from the total set than at high concentration (4.1 ± 2.8 odorants and 8.1 ± 4.7 for methods 1 and 2, respectively, Fig. 5c,d) and a large fraction of cells were in this case not informative for any of the natural odorant tested (9.65% and 27.93% for methods 1 and 2, respectively).

In summary, our data suggest that the breadth of tuning of M/T cells directly reflects the density of glomerular inputs. Indeed, a sparse M/T activation (high lifetime and population sparseness) emerged from low concentrations of natural odorants, probably mimicking observations reported in previous studies^6^,^7^.

## Discussion

In this study we evaluated how natural odorants are represented in the mouse OB and how concentration affects the density of the odor code. By using natural odorant concentrations activating dense glomerular patterns, we showed that M/T cell are more broadly tuned than previously reported, encoding for a large fraction of natural odorants tested (Fig. 3). In contrast, when odorant concentration was reduced, glomerular maps became sparser (Fig. 4) and M/T cell tuning curve narrower (Fig. 5). Altogether, our data support a model of flexible lifetime and population sparseness that depends on, first, the intrinsic natural concentrations and, second, the spike temporal patterning of M/T assemblies.

Previous studies showed that M/T cells recorded in anesthetized animals are highly selective, which was considered to be a consequence of selective OSN activity and odor-specific sparse input maps^6^,^7^,^23^. In contrast, we found in awake mice that M/T cell activity displays a much higher population and lifetime sparseness than previously reported (Fig. 3). Several reasons account for this difference. First, most of the studies were performed using anesthetized mice, which can significantly alter network properties^9^,^24^,^25^. Anesthesia reduces baseline activity and odor-evoked phasic responses of both M/T cells^9^,^25^ and granule cells (GC)^24^, which may consequently reduce the diversity of response patterns in M/T ensembles^11^. Second, until recently, it was generally assumed that a single odorant activated only few glomeruli, as observed with natural odorants^26^. Therefore, previous studies^6^,^7^,^23^ used odorant concentrations that activated only few glomeruli. In contrast, the natural odorants we tested at their native concentrations activated dense glomeruli patterns (Fig. 4), in agreement with our previous report^14^. As a consequence, the M/T cells population and lifetime sparseness observed reflects the density of the input. Supporting this view, natural odorants at lower concentrations, evoking sparser glomerular activation (Fig. 4), increased M/T lifetime and population sparseness (Fig. 5). In this case, M/T cells encoded 4–5 times less odorants than at the intrinsic concentration. Furthermore, previous studies analyzed cell responses by computing rate changes over the respiratory cycle in anesthetized animals^7^,^23^. However, in awake animals, rate changes are not prominent, which has been proposed to support odor encoding with M/T sparse responses^10^,^25^. For example, in many neurons, odors evoke changes in spike timing without changing the average firing rate over a single respiratory cycle^9^,^15^. Consistent with this finding, the majority of M/T cells recorded in our study did not change their firing rate over the complete breath but rather changed their phasic activity across the sniff.

Finally, all previous studies computed M/T cells tuning curves by focusing on single cell response profiles^6^,^7^,^15^,^16^,^23^,^25^. However, we show that a single cell analysis underestimates the actual percentage of neurons contributing to sensory coding (from 28% to virtually all recorded neurons-, Figs 1 and 3). Using a classifying analysis of neuronal ensembles, we observed that even the presumptive “unresponsive” cells were actually encoding odorant information. It is noteworthy that the number of odor trials per session had to be minimized (reduced here to 6) in order to enlarge the panel of natural odorants (29 different stimuli). The limited number of trials along with the baseline variability may lead to a potential bias for extracting single neuron responses. As neural ensembles are also used to efficiently encode sensory information in different species^15^,^27^,^28^,^29^,^30^,^31^,^32^,^33^, we used a population approach to classify informative neurons. This approach turned out to be less sensitive to the low number of trials. Thus, we found that 72–93% of the stimulus set was encoded by our limited number of cells (Fig. 3). The tuning curve of the M/T cell population was much broader (low lifetime and population sparseness) than previously reported^6^,^7^,^23^, with each neuron being informative on average for between 23 to 43% of the natural odorant set.

Two important points must be mentioned. First, when considering odorants that activate a large fraction of neurons, the responsive neurons are not all coactive at a given time; they indeed exhibit complex temporal discharges during the respiratory cycle. Since the odor code is distributed and dynamic in the population of M/T cells^9^,^11^,^13^,^15^,^17^,^27^, only a fraction of the cells are coactive at a given period of time. Second, the M/T neurons appeared to be more broadly tuned than the glomeruli responses. This result is consistent with previous work reporting that projection neurons display a broader tuning than OSNs in the drosophila antennal lobe, hence increasing differences between odor representations^34^. It may also reflect M/T cell modulation by the GABAergic network, much more active in awake animals^24^, which decorrelates^11^,^13^ but also sparsen firing patterns of M/T cells assemblies^10^,^12^,^35^,^36^. Though it appears as an increase of lifetime sparseness, inhibition actually sculpts the temporal patterning of M/T cell activity, presumably improving M/T cell encoding capabilities. Indeed, our method depicts the contribution of both excitatory and inhibitory epochs within a breathing cycle to encode odorant identity.

In conclusion, we found that M/T assemblies are encoding a broad panel of natural odorants at their natural concentrations, processing sensory information with subtle temporal changes of activity.

## Materials and Methods

### Animals and initial preparation

All experiments were performed on 12 to 20 week-old male C57BL/6J mice (Charles River France). All experiments were in accordance with the Swiss Federal Act on Animal Protection and Swiss Animal Protection Ordinance. The experiments were approved by the University of Geneva and the state of Geneva ethics committees (authorization 1007/3387/2).

The initial preparation procedure has been extensively presented elsewhere^9^,^14^,^37^. In brief, a few days before performing experiments, a custom made head-fixation system was mounted on the animal head. A few days after recovery, the animal was placed in a tube and head-fixed by screwing the head-post into a home-made metal device fixed on the air table. Mice were trained in this restrained condition for 2–4 sessions (30 min each) over 2 days. For all habituation tasks and experiments, respiration was monitored using a directional air flow sensor (AWM2100V, Honeywell, MN) placed in front of the mouse snout. This sensor did not occult the mouse nose and thus did not prevent the odor to reach the nostrils.

### Odorant preparation and stimulation

All natural stimuli (banana, honey, ripe kiwi, coffee, oregano, strawberry, Italian cheese (parmesan), Swiss chocolate, wine rosé, nutmeg, rosemary, mint, cinnamon, tobacco, cardamom, cloves, vinegar, garlic, onion, pineapple, basil, lemongrass, apple, pear, sesame oil, black pepper) except mouse food, urine and feces were purchased from local grocery stores. C57BL/6J male mouse urine was collected and stored at −80 °C until use. Mouse feces were collected every day from cages containing male mice. Natural odorants were prepared fresh each experimental day (for example, fruits were freshly pressed and used for a few hours maximum). Either four grams of dry solid (for mouse feces: 1g) or four milliliters (for mouse urine: 1 ml) of natural stimuli were placed in glass vials. They were thus at natural partial pressure.

Through a custom made olfactometer, odorants were delivered during an animal’s expiration (the odor onset was the onset of the first inspiration after delivery) as described previously^9^,^14^,^17^,^19^. For intrinsic optical imaging and tetrode recordings, odorants were delivered for 5 s or 1.5 s, respectively. The number of trials for each odor was 4 and 6 for imaging and electrophysiology, respectively. An air flow passed through the vials containing the odorants. Because odors were delivered ~1 cm away from the animal’s nose, the values overestimate concentrations actually reaching the nasal cavity. The total flow was constant (0.4 l/min). To maintain a stable odor concentration during the entire stimulus application, we ensured that flows were stationary with a 5 s preloading before the odorant was delivered.

### Intrinsic Optical imaging

The procedure has been presented in detail elsewhere^14^,^38^,^39^. In brief, the olfactory bulb was illuminated with red light at 700 nm (BP 20 nm) using a stable 100 W halogen lamp and a light guide system. Images were acquired at 5 Hz for 10 s (2 s before, 5 s during and 3 s after the stimulus) using the Imager 3001F system (Optical Imaging, Mountainside, NJ) mounted both on a custom built macroscope (Navitar 17 mm, bottom lenses, Nikon 135 mm, upper lens; total magnification 7.9x). Images were acquired at 512 × 512 pixels and further 2 × 2 binned. For analyses, all image were band pass filtered (σ1 = 2; σ2 = 100) and realigned by comparing blood vessels patterns between images using a custom script running in Matlab (The MathWorks Inc; Natick, MA).

### *In vivo* electrophysiological recordings and spike sorting

The procedures have been described extensively elsewhere^9^,^27^. In brief, a 1–2 mm window was drilled above the olfactory bulb and the dura mater was opened. One or two silicon-based recording electrodes (A-4 × 2-Tet-5 mm-150-200-312, NeuroNexus Technologies, Ann Arbor, MI, USA) were inserted. Electrodes were lowered vertically in the target zone until the dorsal or medial mitral/tufted cell layer was reached. We used low impedance electrodes (1–4 MΩ at 1 kHz). They underlie stability and reasonable size of the extracellular spikes with respect to the background noise in order to identify single-cell activity after spike sorting. Thus, in the case of low impedances electrodes, these conditions could mainly be fulfilled by M/T cells (the larger cells in the OB), as previously observed by others^6^,^15^,^16^,^25^,^27^. 81 isolated neurons were recorded in 4 mice using natural odorant at high concentration while 79 isolated neurons in 4 additional mice were used for the low concentration natural odorant dataset. All subsequent analyses and statistics were calculated using custom scripts written for Matlab (MathWorks, Inc., Natick, MA) or C.

### Data analysis

#### Breathing cycle realignment

In awake mice, the duration of respiratory cycles (RC) is highly variable within and across trials. In order to consistently analyze the neural responses to odors across trials, the beginning of each cycle was temporally realigned to each other. All RC were artificially matched to the average breathing duration (405 ms ± 178 S.D.) over all trials: longer cycles were truncated and shorter ones were prolonged. Spike times were realigned using the same method. Importantly, relative action potential timings in spike trains were not affected by this method.

#### Analysis of single cell responses

The procedure has been presented in detail elsewhere^9^. In brief, we divided each respiratory cycle into 8 time bins (50 ms) in which we computed the average firing rates. For each trial, we described the firing activity over consecutive respiratory cycles by a 8 × *n* matrix (*n* respiratory cycles before and during odor presentation). For each odor, we further averaged all matrices computed for individual trials. The same process was applied for all the 29 odors and the averaged matrices were concatenated together (total size, 8 × 7*n*). A principal component analysis (PCA) was computed with the concatenated matrix, which allowed representing each respiratory cycle (RC) as a vector in an 8 dimensional space, each dimension representing one of the respiratory bins. The 3 first components of the PCA transformation carried more than ~75% of the variance.

To define if neurons were responsive to odorants, we assessed whether the firing distribution in RC of the baseline and odor periods was significantly different^9^. For that, in the PCA space, we measured the Euclidean distances between RC of the odor and baseline periods. First, the RCs were segregated into baseline respiratory cycles (BRCs) and odor-evoked respiratory cycles (ORCs). The BRC was again divided randomly into two groups with an equivalent number of BRC: the control BRC (CBRC) and the test BRC (TBRC). We then computed the CBRC centroid and the average distance (d_mean_) and the standard deviation (σ) of the Euclidean distances between the CBRC centroid and each of the single CBRC. Then the distances (K_o_) between each single ORC and the CBRC centroid were calculated. A cell was considered as responsive if:


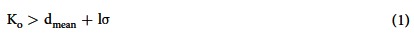


where λ = 1, 2, 3 … n, for at least one odor. However, this method may detect false-positive responses. In order to discard them, we computed the distances (K_b_) between TBRC and the CBRC centroid. Similarly, if:


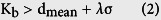


a cell was considered as responsive for at least one odor (where λ = 1, 2, 3 … n). The number λ of standard deviation σ was parametrically increased until only 5% of cells were considered as responsive using the CBRC template. We thus kept the value of λ extracted from the equation (2) and used it to satisfy the conditions in the equation (1). This led to a detection of responsive cells with a risk of false-positive inferior or equal to 5%.

In this analysis, a cell had to respond significantly for at least 3 RCs to be considered as responsive. Similar results were observed by considering one or two significant RCs but the exact percentages of responsive cells were lower due to an increase of false positives in the baseline.

#### Population vector construction and prediction algorithm

We pooled all neurons recorded in different animals, assuming that they represent the same variability of neural responses as an equal number of cells in a single mouse. The activity of the neurons was organized in vectors, each dimension containing the average firing rate of a recorded cell computed over a certain time bin. Population vectors were built using 8 (on average, 50 ms) per breathing cycle time bins.

To compute classification performance, one trial per stimulus was chosen to be a test set and removed from the remaining trials that were averaged and considered as the template responses^9^,^27^. The Euclidean distances between test trials and all stimuli templates were computed, and trials were assigned to the closest template (i.e. to a stimulus prediction). The percentage of success for odor identity and intensity was the fraction of correct assignments over the total number of assignments.

#### Analysis of individual and population of informative cells

In order to identify which individual cells contain information about odorants, one has to determine if odor-evoked activity can be discriminated from baseline activity. To do that, single cell firing rates over a breathing cycle were binned in 8 time windows of equivalent durations (50 ms). Single cell activity was thus represented by series of 8 raw vectors. We then processed our classification algorithm as following. The two first odor-evoked activity vectors after odor onset were extracted and chosen as test vectors for a particular trial (To). An odor-evoked activity template was calculated by computing the centroid of remaining vectors (i.e. except To) over the first and second breathing cycle after odor onset. Similarly, the baseline activity template (test baseline template, Cbt) was computed as the centroid of remaining vectors (i.e. except one vector of the baseline) for the 2 breathing cycles before the odor onset. The Euclidean distances between each To and the 2 centroids (Co and Cbt) were calculated. Trials were assigned to the closest template (i.e, representing the odor-evoked activity prediction).

After recurrently testing each individual cell, the cell leading to the minimal classification performance was selected. Each breathing-related 8-raw activity vector of this cell was then concatenated in the 1^st^ dimension to the corresponding 8-raw activity vector of another cell. This led to a series of 16-raw activity vectors made by the activity of 2 cells during successive breathing cycles. We recurrently varied the identity of the second cell in order to again obtain the minimal performance when using the classification algorithm with each possible 16-raws vector (every possible combination of 2 cells). This process was continued until obtaining a population vector constituted of a total of (n × 8) × 1 (where n = number of cells) concatenated bins, which gave rise finally to a maximum classification performance per odor (black curves in Figs 3a,b and 5a,b).

The most informative cells were concatenated at the end of the process. To select them, we defined a classification performance threshold over which the most informative cells in the population are picked up and below which cells were considered as false positive. To do that, we processed as following. Similarly to To, we extracted a baseline activity test vector (Tb) and computed a second baseline activity template (Cbc) corresponding to two other pre-odor breathing cycles (different from the precedent ones) before odor application. To define a threshold, two methods were then applied. For the first one, the baseline activity vector was computed using the same cell order obtained to build the vector for the precedent process, that is to say for reaching the maximal classification performance. The classification algorithm was applied to obtain a prediction curve (light gray curves in Figs 3a,b and 5a,b). We computed the mean and the standard deviation of this curve (dashed gray lines and gray boxes, respectively in Figs 3a,b and 5a,b). All cells added to the population vector that provided more information than the mean prediction plus 3 standard deviations were selected. This value was set to obtain less than 15% of false positives (14.4%) when comparing two different portions of the baseline by recurrently increasing the minimal informative cells. For the second method, Tb was built like To, that is to say by recurrently changing the cell identity and adding it to the population vector in order to obtain the minimal classification performance (Figs 2,3 and 5, blue curves). The maximum prediction obtained with this ranking was set as a threshold. When tested with their odor related activity (To vs Cbt), cells that brought additional information to this noise prediction threshold were picked up and considered as informative.

This analysis allowed us to extract the fraction of informative cells per odor identity and the fraction of cells per effective odor.

## Additional Information

**How to cite this article**: Gschwend, O. *et al*. Dense encoding of natural odorants by ensembles of sparsely activated neurons in the olfactory bulb. *Sci. Rep.* **6**, 36514; doi: 10.1038/srep36514 (2016).

**Publisher’s note:** Springer Nature remains neutral with regard to jurisdictional claims in published maps and institutional affiliations.

## Supplementary Material

Supplementary Information
