# Supplementary material for: Dense encoding of natural odorants by ensembles of sparsely activated neurons in the olfactory bulb
Source: Sci Rep. 2016 Nov 8;6:36514. doi: 10.1038/srep36514 (PMC5099913; doi:10.1038/srep36514)
Supplement: Supplementary Information [file srep36514-s1.pdf]

Dense encoding of natural odorants by ensembles of sparsely activated neurons in the olfactory bulb

Olivier Gschwend, Jonathan Béroud, Roberto Vincis, Ivan Rodriguez and Alan Carleton

Supplemental Figures

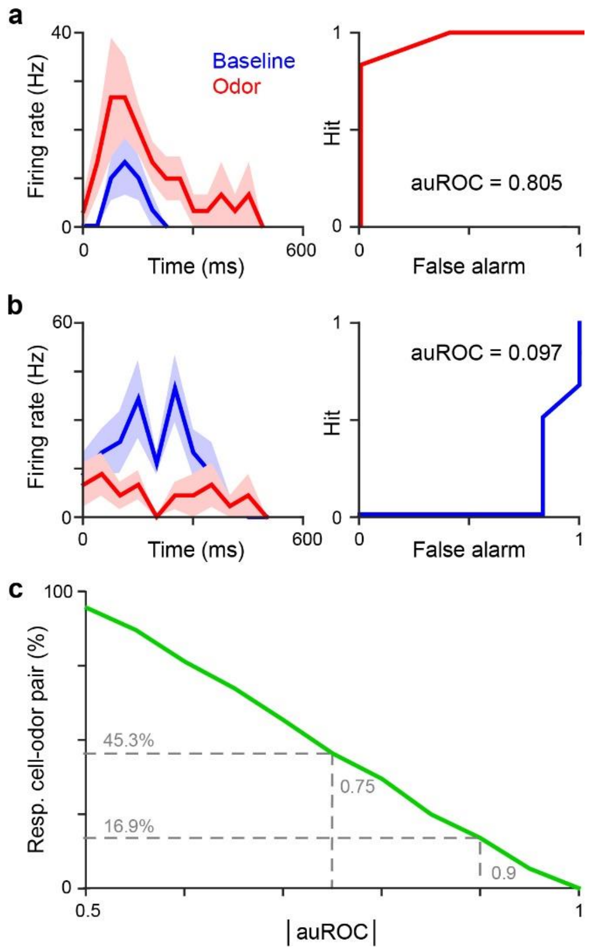

Figure S1. ROC analysis on spiking activity within the first breath after odor onset. (**a**) PSTH of an odor-evoked inhibition of M/T cell firing during the first sniff after odor onset (left panel) and its corresponding ROC curve and auROC (right panel). (**b**) PSTH of an odor-evoked excitation of M/T cell firing during the first sniff after odor onset (left panel) and its corresponding ROC curve and auROC (right panel). (**c**) Percentage of cell-odor pair responses in function of the absolute auROC values. Dashed grey lines indicate the percentage of cell-odor pair responses for auROC absolute values of 0.75 and 0.9.

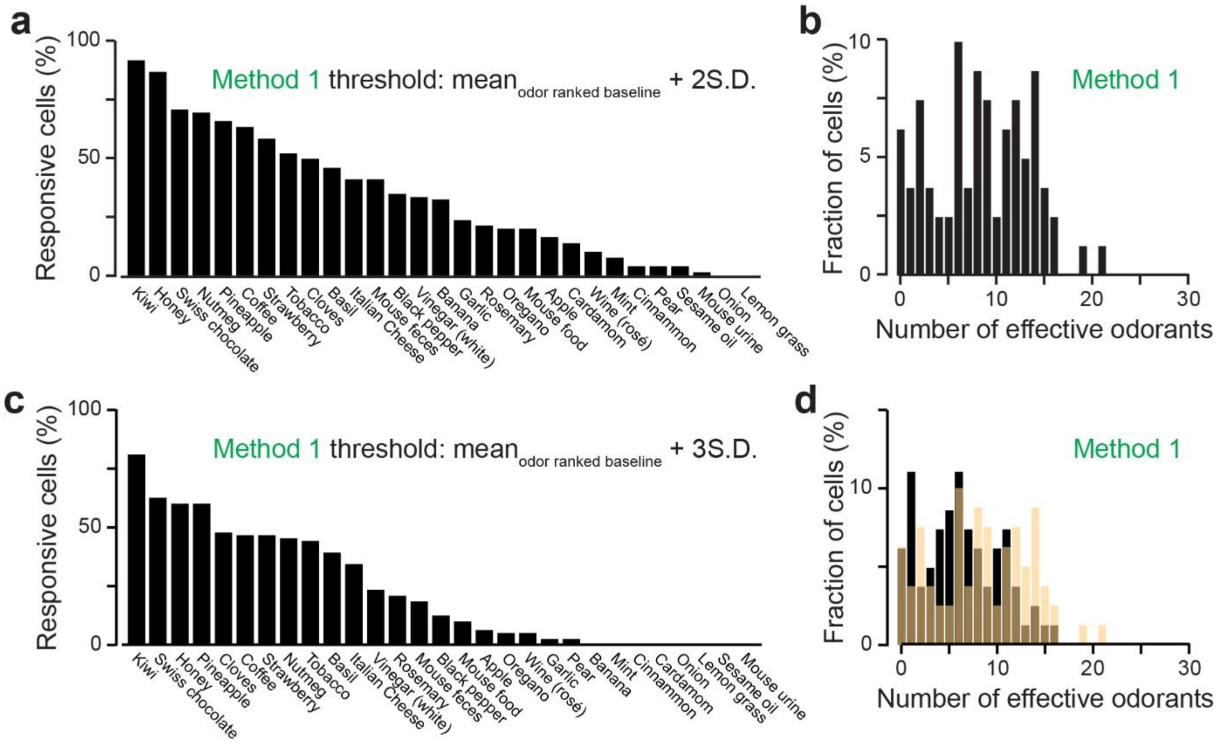

Figure S2. Dependence of M/T cell tuning profiles to the number of standard deviations in the first population coding method. (**a,b**) Fraction of responding cells plotted for each odorant and computed with method 1 using two (**c**) or three S.D. (**d**, same graph as in Fig. 3c). (**b, d**) Fraction of the cell population plotted as a function of the number of effective odorants computed with method 1 using two (**b** and colored histogram in **d**) or three S.D. (**d**, same graph as in Fig. 3c).

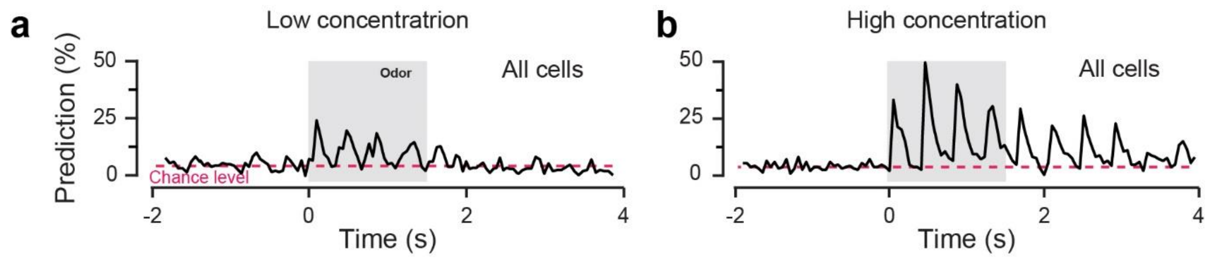

Figure S3. Population prediction performances at different concentrations. (**a**) Prediction (based on the population activity) at low concentrations of natural odorants using the complete cell population and all odorants. (**b**) Similar prediction curves for the dataset at high concentrations (same curve as in Fig. 1c).
